# Factors affecting ozone sensitivity of tobacco Bel-W3 seedlings

**DOI:** 10.1186/1999-3110-54-21

**Published:** 2013-08-27

**Authors:** Ji-Fang Cheng, En-Jang Sun

**Affiliations:** Department of Plant Pathology and Microbiology, National Taiwan University, 10617 Taipei, Taiwan

**Keywords:** Ozone sensitivity, Tobacco Bel-W3, Leaf injury index percentage

## Abstract

**Background:**

Since 1962, the tobacco variety *Nicotiana tabacum* cv. Bel-W3 has been used worldwide as an ozone (O3) bio-indicator. The use of indicator plants to monitor O3 pollution has proven problematic when trying to correlate the severity of injury to ambient O3 concentration. The aim of the present study was to study factors affecting the O3 sensitivity of *Nicotiana tabacum* cv. Bel-W3 seedlings.

**Results:**

In chronic O3 pre-exposure tests, tobacco plants were cultured from seeds in charcoal-filtered air (CF) and noncharcoal-filtered ambient air (NF) for 21 days. During these periods, the mean O3 concentrations of the CF and NF treatments were 5.5 ± 0.2 and 14.7 ± 0.4 ppb h^-1^, respectively (p < 0.001). At the end of the culturing period, no O3-induced foliar injury was observed in any of the plants. The O3 sensitivity of the tobacco plants was determined by exposing the plants to 150 ppb O3 for 8 hours in a fumigation chamber. The leaf injury index percentages (LII%) of indicator plants via the CF and NF treatments were 58.0 ± 3.2% and 43.1 ± 4.0%, respectively (p < 0.01). Twenty-one-day-old tobacco seedlings grown in NF air were used to test the effects of exposed leaves on O3 sensitivity. After removing the cotyledons and all true leaves, the NF seedlings with their apical buds intact were transferred to CF air. After another 7 days of culturing, the newly developed leaves were approximately 1 cm in length. After O3 treatment, the LII% values of the newly developed leaves from the untreated and defoliated seedlings were 2.5 ± 1.7% and 27.6 ± 1.3%, respectively (p < 0.001). In acute O3 exposure tests, 21-day-old seedlings grown in CF air were fumigated with 150 ppb O3 for 8 hours in one day or for 4 hours/day in two consecutive days. The LII% values for the two groups were 63.5 ± 7.4% and 20.1 ± 4.3%, respectively (p < 0.001).

**Conclusions:**

The present findings suggest that plant pre-exposure to O3 is a critical factor influencing O3 sensitivity and that exposed leaves obtain acquired tolerance against O3 injury later on.

**Electronic supplementary material:**

The online version of this article (doi:10.1186/1999-3110-54-21) contains supplementary material, which is available to authorized users.

## Background

The tobacco plant variety *Nicotiana tabacum* cv. Bel-W3 has been used worldwide as an O3-sensitive bio-indicator plant since 1962 (Heggestad 1991). This supersensitive tobacco variety exhibits specific characteristics and foliar responses to ambient levels of O3. The typical symptoms caused by O3 are bi-facial grayish necrotic spots scattered over the lamina (Heggestad 1991; Nali et al. 2006). In threshold studies, the concentration required for the appearance of visible symptoms in seedlings at the age of five leaves, was found to be 80–100 ppb O3 for 4 hours (Heggestad 1991; Sun and Kang 2003). At lower concentrations, the foliar injuries observed in tobacco Bel-W3 seedlings were negligible (Verge et al. 2002).

Although Bel-W3 bio-monitoring systems have been used worldwide (Alves et al. 2011; Calatayud et al. 2007; Heggestad 1991; Klumpp et al. 2006; Lorenzini 1994; Sant'Anna et al. 2008), there is little information about the effects of chronic O3 exposure on Bel-W3. Some studies have shown that pre-exposing Bel-W3 to low doses of O3 predisposes the plant to O3 damage (Heagle and Heck 1974; Steinberger and Naveh 1982). However, in our experience, the indicator plants grown in an O3-free environment are usually more sensitive to O3 than those grown on non-filtered greenhouse benches.

Typically, open-top chamber systems have been used to measure the effects of chronic O3 exposure on plants (Elagoz and Manning 2005; Heagle et al. 1979). Because biotope and climate factors may influence O3 sensitivity (Biondi et al. 1992), we used a patented growth chamber (Sun 1999) to determine the effects of chronic O3 exposure. The growth chamber has an easily renewable filter, and the incoming indoor ambient atmosphere is filtered with active charcoal (CF) or with equally sized inactive particles (NF). To fit the small space of the growth chamber, a miniaturized system for O3 bio-monitoring developed at and patented by the University of Pisa in 1994 (Lorenzini 1994; Nali et al. 2006) was used. Fifteen-day-old tobacco germlings were used as indicators. After 5–7 days of ambient air exposure, the germlings exhibited typical O3-induced foliar injuries. The methodologies of this paper were based on the miniaturized system with some modifications, and 21-day-old tobacco seedlings were used for the experiments.

The aim of this study was to explore the effects of pre-exposure on the O3 sensitivity of tobacco plants using standardized procedures. In addition, the effects of exposed leaves to the O3 sensitivity of the indicators were also studied.

## Methods

### Pre-exposure

Bel-W3 plants were seeded and cultured for 21 days in a patented growth chamber (Sun 1999) with some modifications. The chambers were equipped with renewable filters. In the CF and NF treatments, the incoming air was filtered with activated charcoal particles and with equivalently sized inactive particles, respectively. The O3 concentrations in the growth chamber were measured using an O3 analyzer (Ecotech Model 9810, Pollution Instrumentation, Inc.) at 1-hour intervals. During the 21-day culturing period, the hourly O3 concentrations were used to estimate AOT20 (accumulated O3 exposure over a threshold of 20 ppb h^-1^). The physical conditions in the chambers were as follows: a 12-hour photoperiod, 500 PAR light intensity, 37–45% RH, 25°C during the day and 20°C at night.

### Plant material

Seeds of inbred tobacco Bel-W3 were obtained from National Taiwan University. The seeds were planted and cultured in plastic containers containing a 1:1 mixture of peat and vermiculite for 14 days. The 14-day-old germlings were individually transplanted into the wells of tissue culture plates (14 × 9 cm with 24 round wells, 16 mm in diameter and 20 mm deep) filled with organic compost. The bottoms of the wells were perforated to permit sub-irrigation. After another 7 days of culturing, the 21-day-old seedlings were used for fumigation tests.

### O3 fumigation

The O3 fumigation test was conducted in a controlled chamber (0.9 m × 0.9 m × 1.8 m) equipped with charcoal filters. Before fumigation, the plants were acclimated in the controlled chamber for 24 hours. An O3 generator was adjusted to 150 ppb using an analog voltage regulator. The O3 concentrations were monitored by an O3 analyzer (Thermo Model 49, Thermo Environment Inc.) at 15-minute intervals. The seedlings were exposed to 150 ppb O3 for 8 hours in one day or for 4 hours/day in two consecutive days. Following fumigation, the exposed seedlings were recovered and maintained in CF air for 48 hours to permit the full development of symptoms. The environmental conditions within the chamber were also recorded.

### Visible foliar injury assessment

The percentage of each leaf covered by lesions characteristic of O3 injury was visually determined by comparison with standard pictures. Five classes of injury were distinguished on a percentage basis of the adaxial leaf surface: 0, no lesions; 1, 1–10%; 2, 11–25%; 3, 26–50%; and 4, more than 50% of the area was covered by lesions (Nali et al. 2006).

### New leaf test

Plant materials were cultured in a NF growth chamber as previously described. The control seedlings were cultured in NF air for 3 weeks then transferred to a CF growth chamber for 7 days. For the treatment group, the old leaves of the seedlings were removed, and the plants were subsequently transferred to CF air for 7 days to permit the development of new leaves.

### Nonconsecutive O3 fumigation

Plant materials were cultured in a CF growth chamber as previously described. The control plants were exposed to 150 ppb O3 for 8 hours, while plants in the treatment group were exposed to 150 ppb O3 for 4 hours/day in two consecutive days. After O3 fumigation, the plants were returned to the CF growth chamber.

## Results

**Figure 1:**
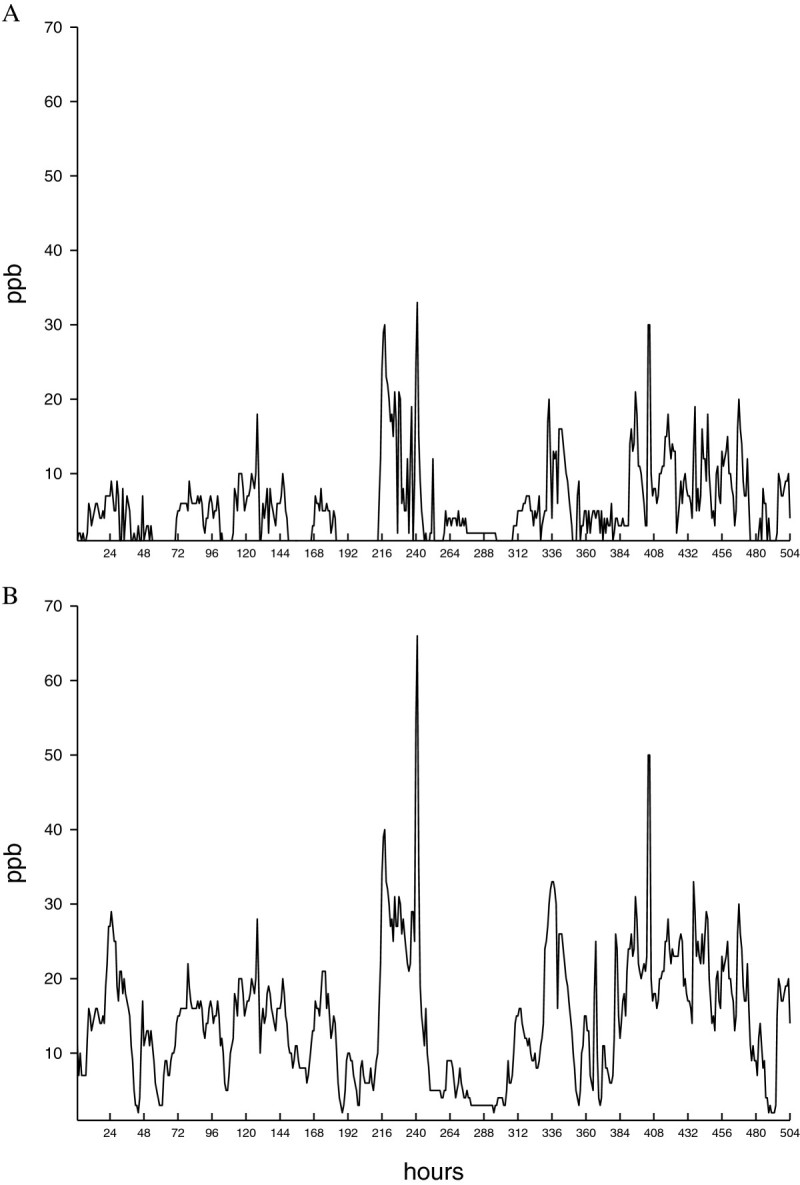
**Dynamic changes of O3 concentrations in the CF and NF growth chambers during the culturing period. (A)** CF treatment and **(B)** NF treatment.

Tobacco Bel-W3 seeds were cultured from March 25, 2013 to April 14, 2013. The changes in the O3 concentrations of the CF and NF treatments during the 21-day period are shown in Figure 1. One peak episode of 66 ppb O3 occurred in the NF treatment on April 3, 2013. The mean O3 concentrations of the CF and NF treatments were 5.5 ± 0.2 and 14.7 ± 0.4 ppb h^-1^, respectively (p < 0.001). At the end of the culturing period, the accumulated O3 exposures above the 20 ppb h^-1^ threshold (AOT20) of the CF and NF treatments were 69 and 770 ppb h^-1^, respectively. No O3-induced foliar injury was observed in any of the plants.

**Figure 2:**
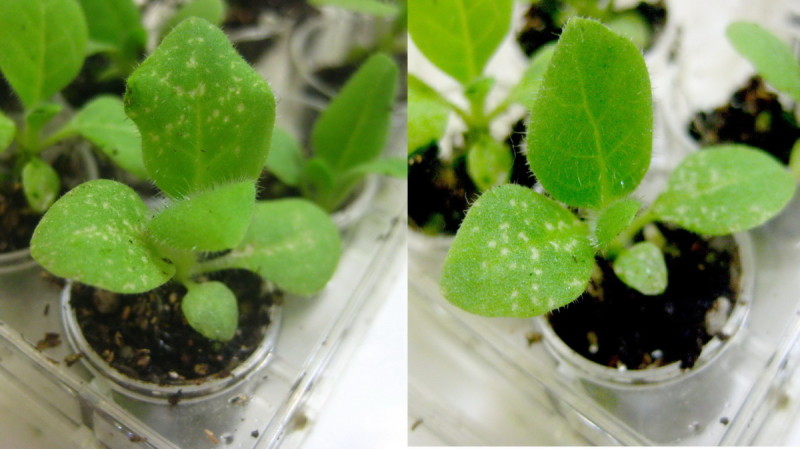
**Chronic ambient O3 exposure attenuated O3-induced foliar injury.** Seedlings cultured in charcoal-filtered air (CF, left) and indoor ambient air (NF, right) for 3 weeks were exposed to 150 ppb O3 for 8 hours. A significant decrease in foliar injury was observed in NF seedlings compared to CF seedlings (p < 0.01, *t*-test).

**Figure 3:**
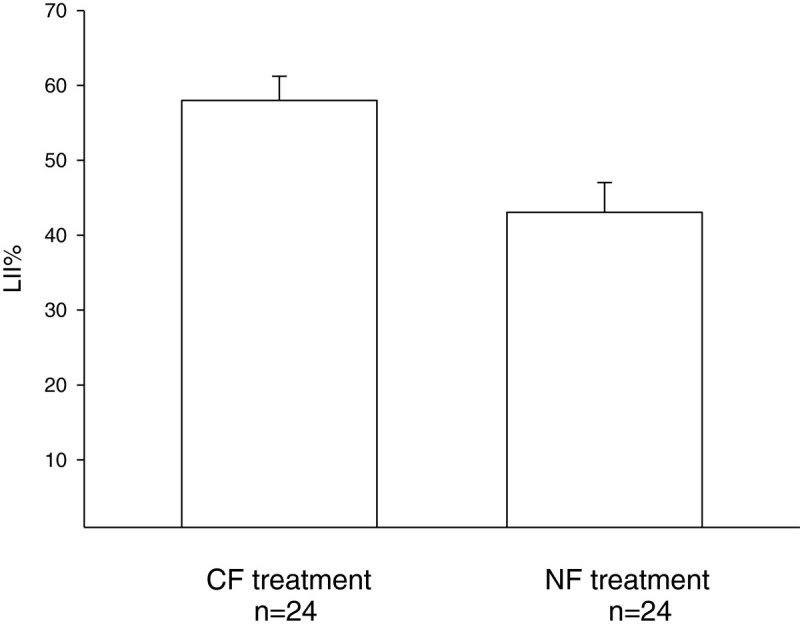
**Chronic ambient O3 exposure reduced the leaf injury index percentage (LII%).** Seedlings cultured in charcoal-filtered air (CF, n = 24) and indoor ambient air (NF, n = 24) for 3 weeks were exposed to 150 ppb O3 for 8 hours in a controlled chamber. The LII% was measured 2 days after exposure. The extent of foliar injury in NF seedlings was significantly decreased compared to that in CF seedlings (p < 0.01, *t*-test).

The tobacco plants that had been exposed to 150 ppb O3 for 8 hours as seedlings exhibited typical O3-induced injury (Figure 2). The leaf injury index percentages (LII%) of the CF and NF treatments were 58.0 ± 3.2% and 43.1 ± 4.0%, respectively (p < 0.01, *t*-test) (Figure 3). The results indicate that tobacco seedlings cultured in CF air were more sensitive to O3 than those cultured in ambient air.

**Figure 4:**
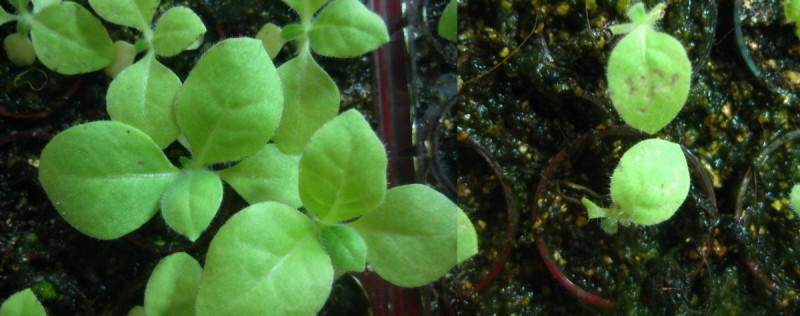
**O3-exposed leaves protected new leaves against the effects of subsequent O3 exposure.** Seedlings cultured in indoor ambient air for 3 weeks were transferred to a charcoal-filtered chamber. After 7 days, the newly developed leaves grew to a length of approximately 1 cm. A significant difference in the severity of foliar injury was observed for newly developed leaves with (left) and without (right) old leaves after exposure to 150 ppb O3 for 8 hours.

**Figure 5:**
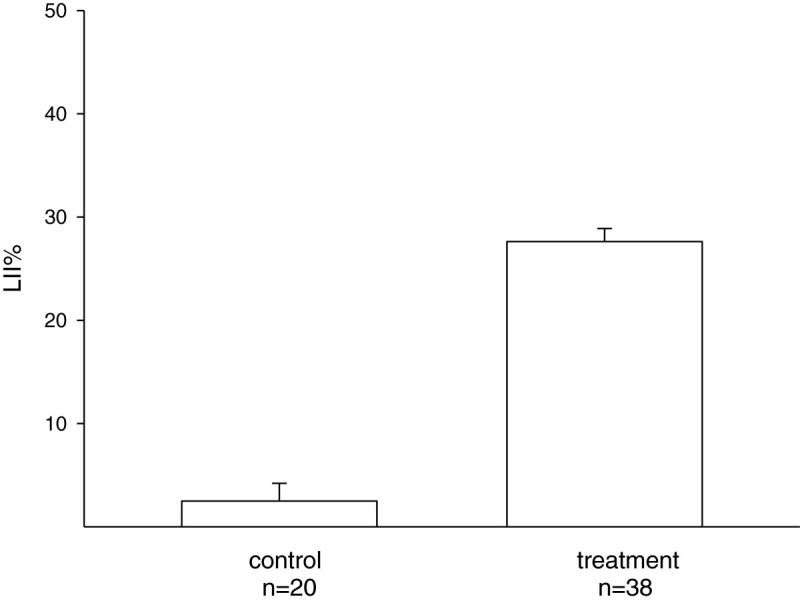
**O3-exposed leaves protected new leaves against the effects of subsequent O3 exposure.** Control seedlings were cultured in indoor ambient air (NF, n = 20) for 3 weeks then transferred to a charcoal-filtered chamber. For the treatment, 21-day-old seedlings were grown in NF air, defoliated, and then transferred to CF air for 7 days to allow new foliage to grow (n = 38). There was a significant difference in the leaf injury index percentages (LII%) of seedlings with and without old leaves after exposure to 150 ppb O3 for 8 hours (p < 0.001).

In the experiment in which 21-day-old seedlings grown in NF air were defoliated, the plants were transferred to CF air for 7 days to permit the development of new foliage. The newly developed leaves grew to a length of approximately 1 cm (Figure 4). After exposure to 150 ppb O3 for 8 hours, the LII% values of the newly developed leaves from the untreated and defoliated seedlings were 2.5 ± 1.7% and 27.6 ± 1.3%, respectively (P < 0.001) (Figure 5). The results indicate that the newly developed leaves of the defoliated plants were more sensitive to O3.

**Figure 6:**
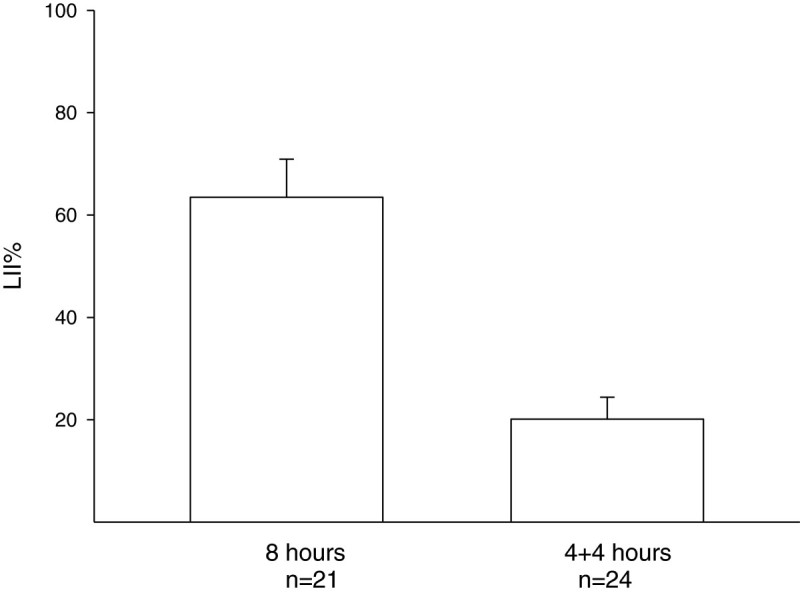
**Under the same dosage of O3 exposure, seedlings exposed for 8 hours in one day were more sensitive than seedlings exposed for 4 hours/day in two consecutive days.** There was a significant difference between the leaf injury index percentages (LII%) of seedlings exposed to 150 ppb O3 for 8 hours in one day (n = 21) and of those exposed for 4 hours/day in two consecutive days (n = 24, p < 0.001, *t*-test).

In another experiment, 21-day-old seedlings were grown in CF air then exposed to 150 ppb O3 for 8 hours in one day or for 4 hours/day in two consecutive days. The LII% values for the two groups were 63.5 ± 7.4% and 20.1 ± 4.3%, respectively (p < 0.001, *t*-test). The results showed that for the same level of O3 exposure, the seedlings subjected to the longer one-day exposure were more sensitive than those whose total O3 exposure was divided over two consecutive days (Figure 6).

## Discussion

The results of the present study show that low doses of O3 exposure for long periods attenuate the O3 sensitivity of tobacco Bel-W3 seedlings. Steinberger and Naveh (1982) showed that exposing Bel-W3 tobacco to 30 ppb O3 for 12 hours did not cause any visible injury. However, the exposed plants were more sensitive to subsequent O3 exposure than untreated plants. In that experiment, the tobacco seeds were grown in a charcoal-filtered environment prior to the tests. Pre-exposure of plants to O3 may therefore be a critical factor influencing O3 sensitivity. Additionally, the present study showed that the exposed leaves of seedlings protected them against the effects of subsequent O3 exposure.

The use of indicator plants to monitor air pollutants has proven problematic when trying to correlate the severity of injury to ambient O3 concentration (Heck et al. 1966; Larsen et al. 1993). The possible mechanisms underlying the non-linear O3 damage of Bel-W3 tobacco have been considered. For example, it was previously reported that O3 pre-exposure predisposes Bel-W3 to O3 damage (Nali et al. 2006). In the present study, we propose another mechanism that may explain the non-linear relationship: exposed older leaves may influence the performance of the bio-indicator. Therefore, we suggest that old leaves be removed during the monitoring period to ensure that the O3 sensitivity of the tobacco plants is elevated. The roles of exposed older leaves in the O3 sensitivity of Bel-W3 seedlings will be characterized in our future studies.

Because genomic factors also affect the response of bio-indicators, we previously developed simple procedures for culturing Bel-W3 plant tissues in test tubes (Sun and Kang 2003). In our pre-tests, the tobacco plantlets acclimatized in CF air were more sensitive to O3 than those acclimatized in NF air. With tissue culture, plantlets grown in vitro must acclimatize to their environments after being transferred to open air, and the sudden increase in O3 concentration may critically affect O3 sensitivity. The tissue culture system provides an ideal model for studying the effect of ambient air on O3 sensitivity in future studies.

## Conclusions

Pre-exposure of O3 is a critical factor influencing O3 sensitivity of the tobacco Bel-W3 seedlings. The exposed leaves acquire resistance against subsequent O3 exposures. The results may explain no-linear relation between O3 dose and injury to the tobacco Bel-W3.

## Authors’ original submitted files for images

Below are the links to the authors’ original submitted files for images. Authors’ original file for figure 1 Authors’ original file for figure 2 Authors’ original file for figure 3 Authors’ original file for figure 4 Authors’ original file for figure 5 Authors’ original file for figure 6
