# Supplementary figures and images for: Factors affecting ozone sensitivity of tobacco Bel-W3 seedlings
Source: Bot Stud. 2013 Aug 27;54:21. doi: 10.1186/1999-3110-54-21 (PMC5432737; doi:10.1186/1999-3110-54-21)

A

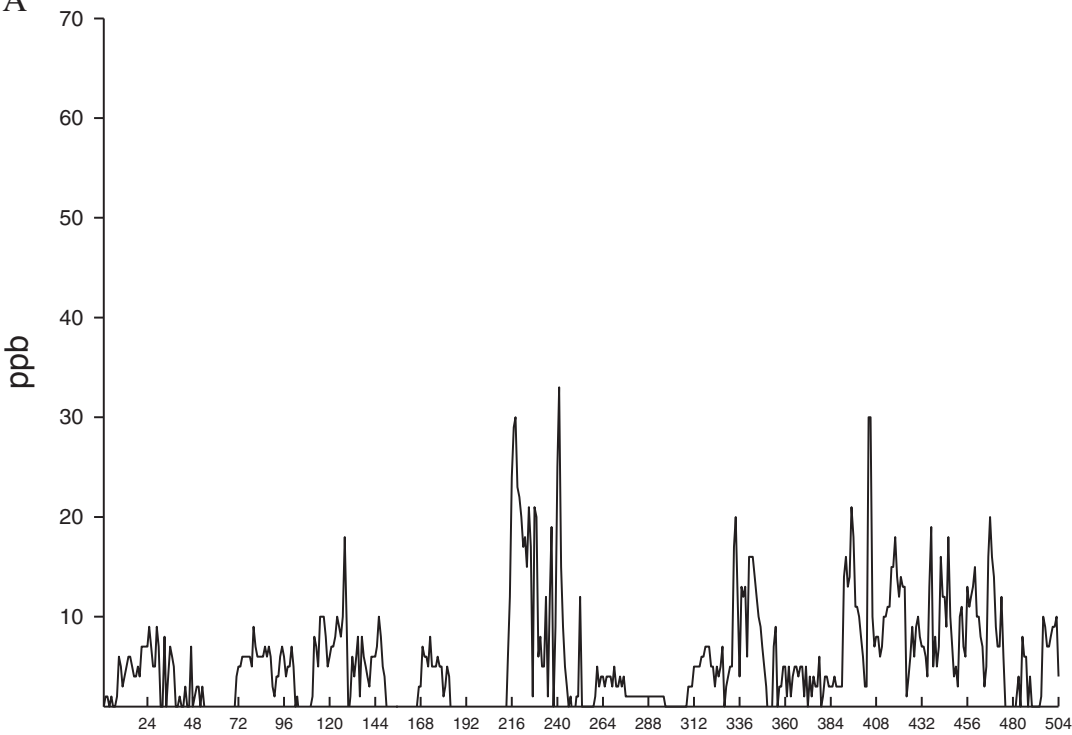

B

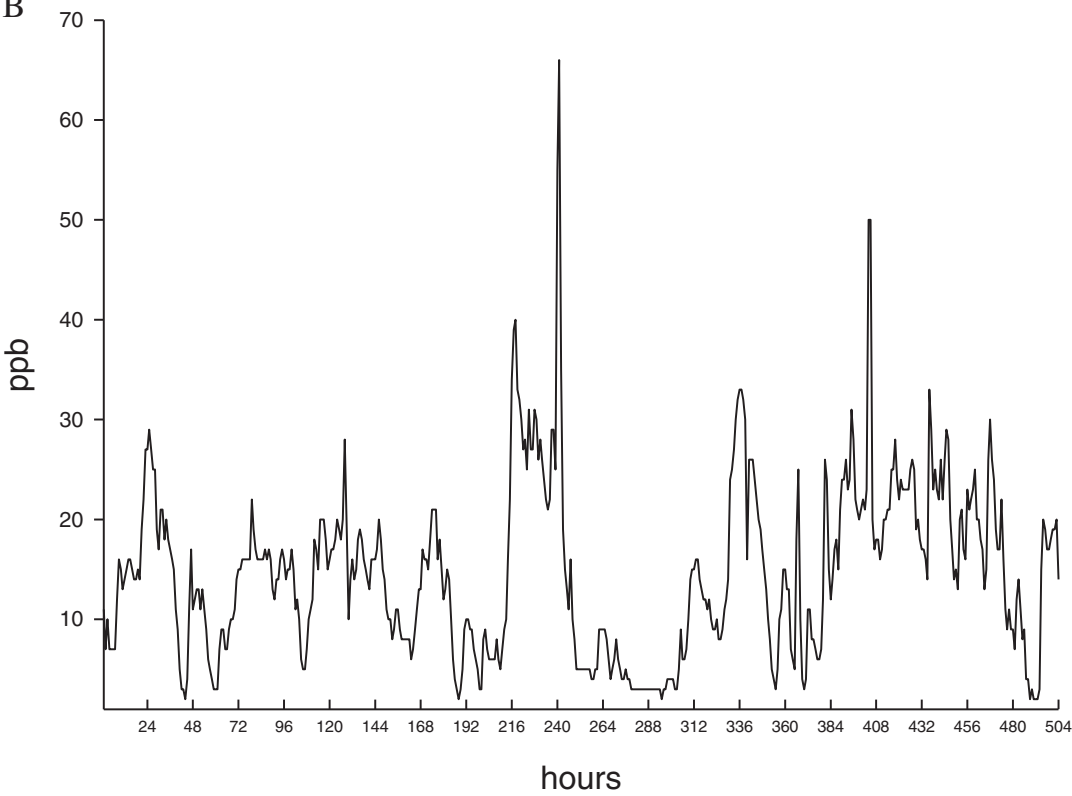

Supplement: Supplementary file 1 — Authors’ original file for figure 1 [file 40529_2013_27_MOESM1_ESM.pdf]

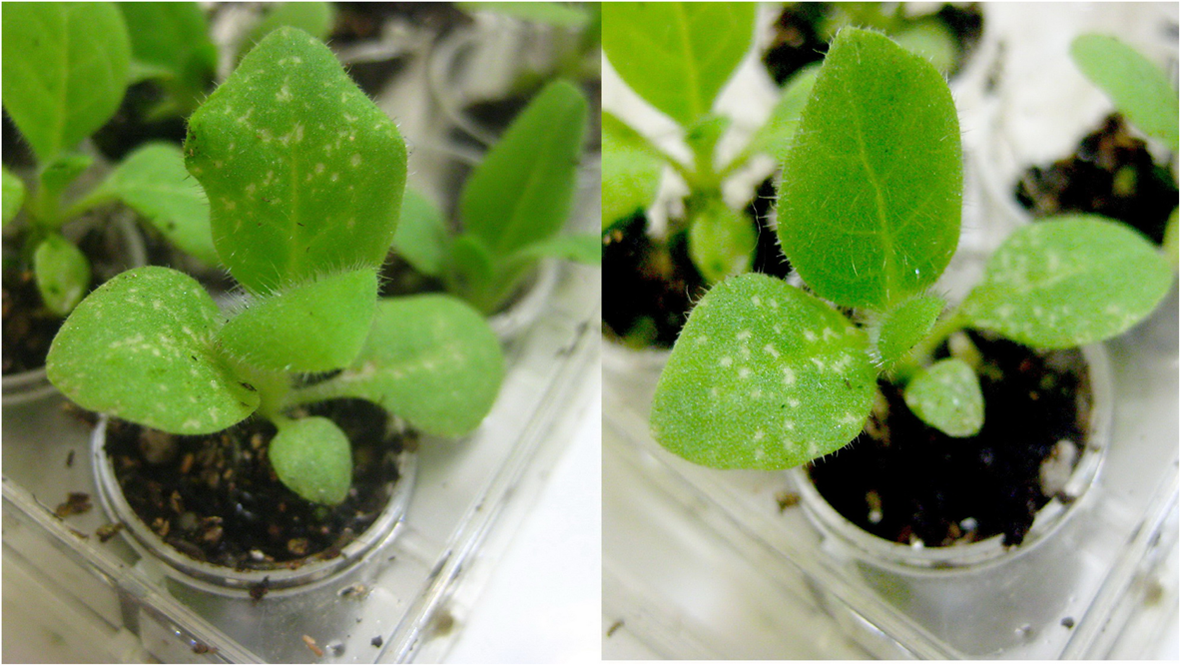

Supplement: Supplementary file 2 — Authors’ original file for figure 2 [file 40529_2013_27_MOESM2_ESM.tif]

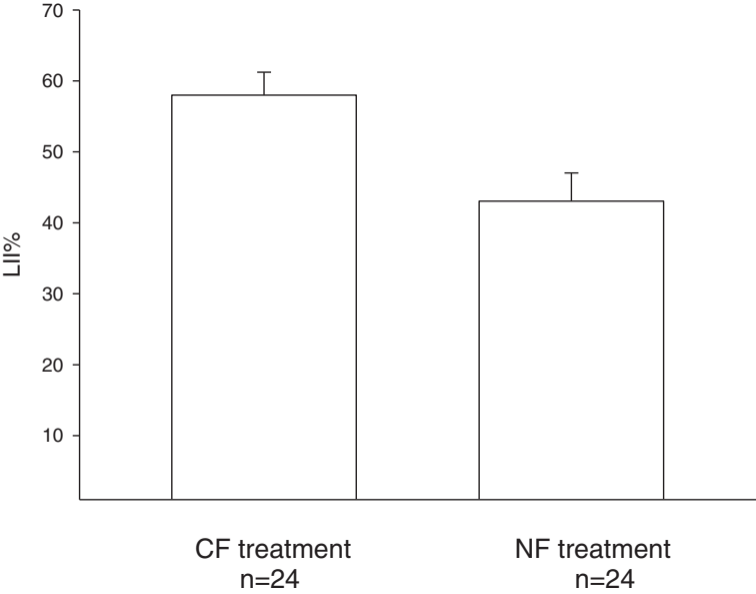

Supplement: Supplementary file 3 — Authors’ original file for figure 3 [file 40529_2013_27_MOESM3_ESM.pdf]

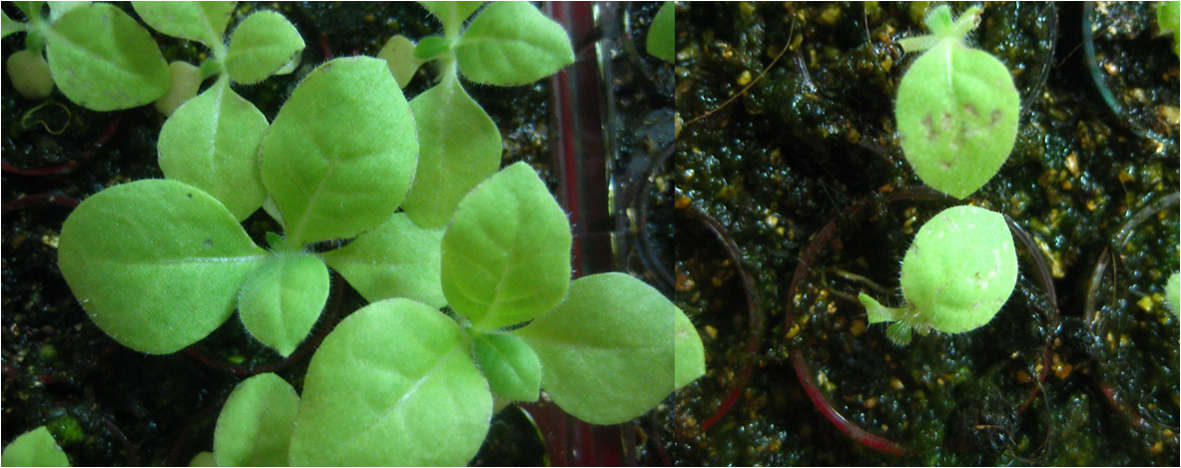

Supplement: Supplementary file 4 — Authors’ original file for figure 4 [file 40529_2013_27_MOESM4_ESM.tif]

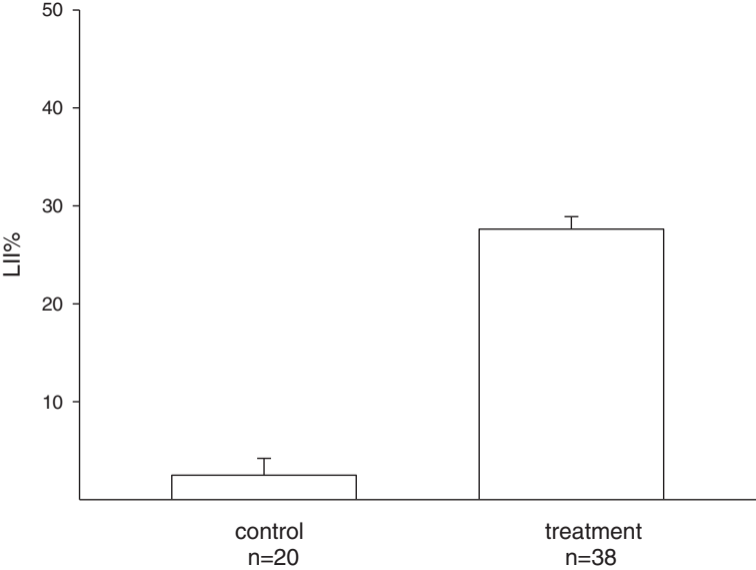

Supplement: Supplementary file 5 — Authors’ original file for figure 5 [file 40529_2013_27_MOESM5_ESM.pdf]

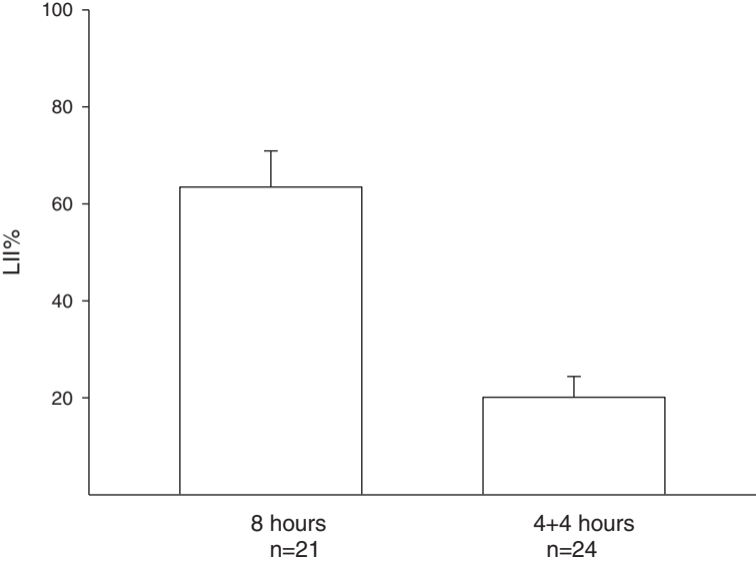

Supplement: Supplementary file 6 — Authors’ original file for figure 6 [file 40529_2013_27_MOESM6_ESM.pdf]
